# Molecular Phylogeny Supports Repeated Adaptation to Burrowing within Small-Eared Shrews Genus of *Cryptotis* (Eulipotyphla, Soricidae)

**DOI:** 10.1371/journal.pone.0140280

**Published:** 2015-10-21

**Authors:** Kai He, Neal Woodman, Sean Boaglio, Mariel Roberts, Sunjana Supekar, Jesús E. Maldonado

**Affiliations:** 1 Department of Vertebrate Zoology, National Museum of Natural History, Smithsonian Institution, Washington, D.C., United States of America; 2 Kunming Institute of Zoology, Chinese Academy of Sciences, Kunming, Yunnan, China; 3 USGS Patuxent Wildlife Research Center, National Museum of Natural History, Smithsonian Institution, Washington, D.C., United States of America; 4 Center for Conservation and Evolutionary Genetics, Smithsonian Conservation Biology Institute, National Zoological Park, Washington, D.C., United States of America; 5 Department of Biology, Pacific Lutheran University, Tacoma, Washington, United States of America; 6 University of Pennsylvania, Philadelphia, Pennsylvania, United States of America; 7 Department of Biology, Oberlin College, Oberlin, Ohio, United States of America; Sichuan University, CHINA

## Abstract

Small-eared shrews of the New World genus *Cryptotis* (Eulipotyphla, Soricidae) comprise at least 42 species that traditionally have been partitioned among four or more species groups based on morphological characters. The *Cryptotis mexicana* species group is of particular interest, because its member species inhibit a subtly graded series of forelimb adaptations that appear to correspond to locomotory behaviors that range from more ambulatory to more fossorial. Unfortunately, the evolutionary relationships both among species in the *C*. *mexicana* group and among the species groups remain unclear. To better understand the phylogeny of this group of shrews, we sequenced two mitochondrial and two nuclear genes. To help interpret the pattern and direction of morphological changes, we also generated a matrix of morphological characters focused on the evolutionarily plastic humerus. We found significant discordant between the resulting molecular and morphological trees, suggesting considerable convergence in the evolution of the humerus. Our results indicate that adaptations for increased burrowing ability evolved repeatedly within the genus *Cryptotis*.

## Introduction

**Fig 1:**
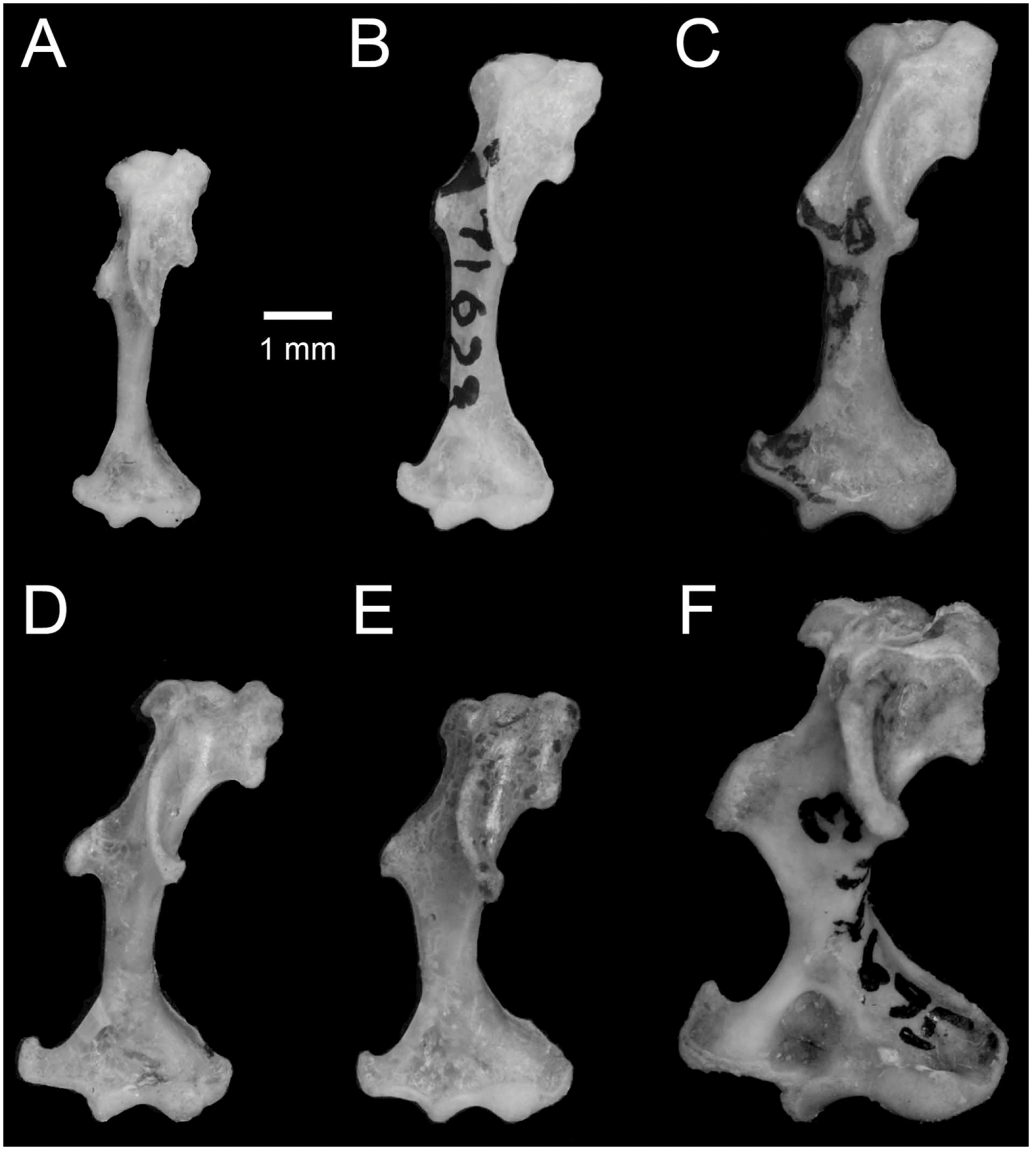
Anterior aspects of left humeri of small-eared shrews, illustrating the range of interspecific variation. (A) *Cryptotis parvus* (*C*. *parvus* group); (B) *C*. *nigrescens* (*C*. *nigrescens* group); (C) *C*. *meridensis* (*C*. *thomasi* group); (D) *C*. *mexicanus* (*C*. *mexicanus* group); (E) *C*. *gracilis* (*C*. *mexicanus* group); (F) *C*. *lacertosus* (*C*. *goldmani* group).

The genus *Cryptotis* [1] is a New World clade of at least 43 species of small-eared shrews (Eulipotyphla, Soricidae) that are discontinuously distributed from the eastern United States and southernmost Canada to the Andes of northern Peru [2–5]. Species have traditionally been divided among four or more species groups that were initially based on external and cranial features (Table 1; [2–4]) and have been further refined using postcranial characters, particularly the morphology of the humerus (Fig 1; e.g., [5]) and the manus [6, 7]. Postcranial characters showed, for example, that a group of so-called “relict species,” a polyphyletic group associated on the basis of their possession of supposedly primitive cranial characters [4], actually belong to other established groups (Table 1). In addition to their morphological similarity, members of individual species groups tend to have similar geographical and elevational distributions that may correspond to distinct physiological limitations, and they exhibit consistent patterns of substrate use and locomotory behavior [2–5].

**Table 1:** Traditional morphologically-defined species groups of small-eared shrews *(Cryptotis*).

species group	description	Species
***Cryptotis nigrescens* group** (*n* = 8)	This group is comprised of species formerly considered subspecies of *C*. *nigrescens*. Species are generally distributed at low to middle elevations and occur from southern Mexico to northern Colombia [2, 4]. This group was once considered part of the *C*. *parvus* group [4]. Species have humeri that are simple in form that are interpreted as reflecting a more ambulatory mode of locomotion (Fig 1; [9]).	*C*. *brachyonyx*, *C*. *colombianus*, *C*. *hondurensis*, *C*. *lacandonensis*, *C*. *mayensis* ^, *C*. *merriami* *, *C*. *merus* and *C*. *nigrescens* *
***Cryptotis parvus* group** (*n* = 6)	Species in this group previously were treated as subspecies of *C*. *parvus*. They generally occur at low to middle elevations from northeastern North America to central Costa Rica. This group formerly included the species now separated out as the *C*. *nigrescens* group [2, 4]. The humerus is simple in form, and it is interpreted as reflecting a more ambulatory mode of locomotion (Fig 1; [9])	*C*. *berlandieri*, *C*. *orophila*, *C*. *parvus* *, *C*. *pueblensis*, *C*. *soricina* and *C*. *tropicalis* *
***Cryptotis mexicanus* group (*sensu lato*)** (*n* = 15)	In its broadest sense, this group includes species previously considered populations and subspecies of *C*. *mexicanus*, *C*. *goldmani*, and *C*. *goodwini* [4, 5] as well as two “relict species” [4], *C*. *gracilis* and *C*. *magnus* (e.g., [10]). These species generally occur at middle to high elevations from eastern Mexico to northern Panama. These species have humeri that exhibit increased robustness, moderate to extreme development of bony processes, and elongation of the humeral head. These features are interpreted as indicating increasingly more fossorial behavior (Fig 1; [9]). Based on our current study, the *C*. *goldmani* group and *C*. *gracilis* are not closely related to other members of the *C*. *mexicanus* group, so the *C*. *mexicanus* group should be restricted to just the first five species listed.	*C*. *magnus* ^, *C*. *mexicanus* *, *C*. *nelsoni* ^, *C*. *obscurus* ^, *C*. *phillipsii* *, as well as members of the *C*. *goldmani* group (see below)
***Cryptotis goldmani* group** (*n* = 10)	This group of broad-clawed shrews was original conceived as a subgroup within the *Cryptotis mexicanus* group (*sensu lato*), and it comprised those species with the most extreme development of fossorial characteristics of the humerus (Fig 1; [5]). Based on our study, the *C*. *goldmani* group, together with *C*. *gracilis*, appear to be in a separate clade from other members of the *C*. *mexicanus* group.	*C*. *alticola* *, *C*. *goldmani* ^, *C*. *goodwini* ^, *C*. *griseoventris*, *C*. *lacertosus* *, *C*. *magnimanus*, *C*. *mam* *, *C*. *oreoryctes* *, *C*. *peregrinus* *, as well as *C*. *gracilis* *.
***Cryptotis thomasi* group** (n = 14)	This group includes the “relict species” [4] *C*. *endersi* and species formerly considered subspecies of *C*. *thomasi*. Members of the group occur at middle to high elevation in Panama and northern South America from Venezuela to northern Peru [3]. The humerus typically is large and robust with a morphology intermediate between the simpler humeri of the *C*. *nigrescens* group and the more robust and complex humeri of the *C*. *mexicanus* group (Fig 1; [9]).	*C*. *aroensis*, *C*. *endersi*, *C*. *equatoris*, *C*. *medellinius*, *C*. *meridensis*, *C*. *montivagus*, *C*. *niausa*, *C*. *osgoodi*, *C*. *perijanensis*, *C*. *peruviensis*, *C*. *squamipes*, *C*. *tamensis*, *C*. *thomasi*, and *C*. *venezuelensis*.

*: species included in both of our genetic analyses and morphological analyses;

^: species only included in the genetic analyses.

Of particular interest has been the *C*. *mexicanus* group (*sensu lato*; Table 1). This group has a broad distribution in Mexico and northern Central America, where its constituent species sometimes occur sympatrically with members of the *C*. *nigrescens* and/or *C*. *parvus* groups. In addition to being a speciose group, the *C*. *mexicanus* group exhibits a range of forelimb morphologies (Fig 1; [8]) that appear to be adaptations for a broad diversity of substrate use and locomotory behaviors distinct from those of the other species groups [9]. Initial phylogenetic analyses of limited morphological transition series focusing on the *C*. *mexicanus* group seemed to support its monophyly and suggested a simple bifurcating pattern of evolution with individual species sequentially diverging from a single primary lineage [5, 6, 10, 11]. Larger datasets from a greater diversity of species, however, indicate that most postcranial characters form a continuum representing a subtly graded series of adaptations from more ambulatory to more fossorial and that evolutionary relationships among the larger sample of species may exhibit more complex pattern of branching [9].

While the putative species groups served as a convenient starting point for studying species diversity [2, 5], biogeography [4, 10], and functional morphology [6, 9], a more comprehensive understanding of evolution in this genus has been hampered by the lack of a robust phylogeny of these groups that is independent of morphology. Unfortunately, most previous molecular phylogenetic studies that included *Cryptotis* were focused on understanding the relationship of the genus within the family Soricidae [12, 13]. These studies generally incorporated few species and are of little assistance in understanding evolutionary relationships within the genus. More recently, Guevara and Cervantes analyzed variation in the mitochondrial cytochrome *b* gene among a large selection of Mexican *Cryptotis*, including members of the *C*. *parvus* and *C*. *mexicanus* groups (Table 1; [14]). Their results supported recognition of the *C*. *parvus* group, but suggested that one or both of the *C*. *mexicanus* and *C*. *goldmani* groups could be paraphyletic. Either circumstance would indicate that evolution of the forelimb skeleton has been more complex within *Cryptotis* than previously realized. Unfortunately, basal node support in the phylogenetic analysis from that study was too low to be confident of the evolutionary relationships of the higher taxonomic groups.

In our study, we sequenced two mitochondrial genes (cytochrome *b*, 16S rRNA) and two nuclear genes (*BRCA1*, *ApoB*) to reconstruct a more robust phylogeny for *Cryptotis*. We also coded 12 morphological characters primarily from the humerus to test the utility of postcranial data used in concert with molecular data. We analyzed morphological and molecular datasets to test the monophyly of species groups that were originally proposed exclusively on morphological characters. We also investigate the role that convergence may have played in the evolution of morphologically similar humeri among phylogenetically divergent groups.

## Materials and Methods

### Ethics statements

No animals were harmed in the execution of this study. All tissue samples sequenced for this study are from specimens previously reposited in the Department of Vertebrate Zoology, National Museum of Natural History, Washington, DC.

### DNA sequences

DNA samples were obtained from preserved tissue samples and from voucher specimens of 3 *Blarina brevicauda* and 33 specimens of 11 species of *Cryptotis* from Guatemala, Costa Rica, Mexico, and the United States (S1 Table). Genomic DNA was extracted using the Qiagen DNeasy Kit (Qiagen Inc., USA). Two mitochondrial genes (16S rRNA and cytochrome *b* [*CYT B*]) and two nuclear genes (Apolipoprotein B [*APOB*] and breast cancer type 1 susceptibility protein [*BRCA1*]) were amplified using previously published [12, 15] and originally designed primers (S2 Table). PCR products were purified and then sequenced using an ABI PRISM 3130 Genetic Analyzer. We sequenced forward and reverse strands of each sample using PCR primers. Sequencher v5.0 was used to edit the sequences. Additional sequences from 3 *Blarinella*, 2 *Blarina* and 36 *Cryptotis* specimens were downloaded from GenBank and included in the final data set. We aligned each gene separately using MUSCLE [16]. The amino acid sequences of coding genes were checked by eye.

### Morphological characters

A total of twelve transition series (skull size and 11 humerus transition series) were coded from 22 species or populations of *Cryptotis*, as well as two outgroups, *Blarinella quadraticauda* and *Blarina brevicauda* (Table 2; S1 Text). Of these, we analyzed only the 17 taxa (including outgroups) for which the character matrix was most complete (i.e., at least 4 characters). One population included in this analysis, listed as “*C*. ‘*goodwini*’ Chiapas”, is a *C*. *goodwini*-like shrew (see [6–9]) from Chiapas, Mexico, that is probably an undescribed member of the *C*. *goldmani* group (L. Guevara, pers. comm.). We do not include this taxon in Table 1.

**Table 2:** Morphological character matrix. Abbriviations of characters are given S1 Text. Letters after species names refer to geographic populations: AV, Alta Verapaz, Guatemala; CH, Chiapas, Mexico; KS, Kansas; MD, Maryland; OAX, Oaxaca, Mexico; PAN, Panama; VA, Virginia; VC, Veracruz, Mexico; Z, Zacapa, Guatemala.

	1	2	3	4	5	6	7	8	9	10	11	12
Species/populations	CBL	HLR	HEB	HRI	HTI	TTP	SHH	trough	HME	pectoral ridge	HH	PP
*Blarinella quadraticauda*	2	1	1	0	1	3	2	0	0	2	1	0
*Blarina brevicauda*	6	2	2	2	1	4	2	0	0	2	1	1
*C*. *alticola*	3	?	?	?	?	?	2	1	?	1	?	?
*C*. *goldmani*	3	?	?	?	?	?	?	?	?	?	?	?
*C*. *goodwini* (CH)	5	2	4	3	2	4	?	?	1	0	?	?
*C*. *gracilis*	2	0	2	1	0/1	3	1 / 2	0	1	2	1	1
*C*. *lacandona*	3	1	0	0	0	1/2	0	0	0	3	?	?
*C*. *lacertosus*	4	1	4	3	2	4	3	1	1	1	2	3
*C*. *magnus*	6	?	?	?	?	?	?	?	?	?	?	?
*C*. *mam*	3	1	3	3	2	4	3	1	1	1	2	1
*C*. *mayensis*	2	?	?	?	?	?	?	?	?	?	?	?
*C*. *merriami* (AV)	3	1	0	0	0	1	0	0	0	3	0	1
*C*. *merriami* (Z)	3	1	0	0	0	0	0	0	0	3	1	0
*C*. *mexicanus* (OAX)	1 / 2	?	?	?	?	?	?	?	?	?	?	?
*C*. *mexicanus* (VC)	2	1	2	1	1	4	2	1	1	1	1	1
*C*. *nelsoni*	2 / 3	?	?	?	?	?	?	?	?	?	?	?
*C*. *nigrescens* (PAN)	2	1	0	0	0	1	0	0	0	3	0	1
*C*. *obscurus*	1 / 2	?	?	?	?	?	?	?	?	?	?	?
*C*. *oreoryctes*	4	1	3	3	2	4	3	1	1	1	1	2
*C*. *parvus* (KS)	0	1	0	0	0	1/2	1	0	0	3	0	1
*C*. *parvus* (MD VA)	0	?	?	?	?	?	0	0	0	3	?	?
*C*. *peregrinus*	2	?	?	?	?	?	?	?	?	?	?	?
*C*. *phillipsii*	2 / 3	1	2	1	1	2	2	0	1	2	1	1
*C*. *tropicalis*	1	1	0	0	0	2	0	0	0	2	1	0

### Phylogenetic analyses

For the DNA sequences, we performed Maximum Likelihood (ML) and Bayesian phylogenetic analyses using RAxML v8.1.11 [17] and BEAST v1.8.1 [18], respectively. We used PartitionFinder v1.1.1 [19] with two strategies to determine the best-fit partitioning schemes and evolutionary models for each partition under the Bayesian Information Criterion (BIC). We first concatenated the four genes and defined data blocks based on genes and coding positions and used the Greedy Heuristic Algorithm to search the best partition scheme. Secondly, we ran PartitionFinder for each gene separately and defined data blocks based on coding positions. We limited the models to GTR and GTR+Gamma for RAxML, because the software did not allow any other models. The first strategy found a 5-partition scheme for BEAST and a 4-partition scheme for RAxML. When using the second strategy, we found a 7-partition scheme for BEAST and a 6-partition scheme for RAxML. Partition schemes and evolutionary models are provided in S3 Table. We used the partitioning schemes determined by both strategies to analyze concatenated species trees. We also estimated the mitochondrial and each nuclear gene tree using the results of the second strategy.

The RAxML analyses were performed on the CIPRES Science Gateway platform [20]. We ran 500 bootstrap replicates using the rapid bootstrapping algorithm. For BEAST analyses, we used a random starting tree, a lognormal relaxed molecular clock and birth-death tree prior. The CTMC model was selected as the prior for evolutionary rate [21]. We repeated the analyses twice. Each analysis was run for 50 million generations and sampled every 5,000 generations. Trace v1.6 was used to ensure the Markov chains had reached stationary states (i.e., effective sample sizes > 200).

We used Maximum Parsimony (MP) to analyze the morphological data. We implemented MP analyses using PAUP 4.0b10 [22]. The characters were optimized using “delayed transformation” on the trees in memory (opt = DELTRAN). We performed heuristic searches with 10,000 random addition replicates using the TBR branch-swapping algorithm. MP bootstrap values were calculated from 100 replicates of random addition sequence. All morphological characters were weighted equally. We performed four morphological analyses. First, we constrained the topology of the outgroups as (*Blarinella*, (*Blarina*, *Cryptotis*)), and analyzed the data set with transformation series unordered. Secondly, based on the results of molecular phylogenetic analyses (see Results), we constrained the highly supported relationships (i.e., posterior probabilities ≥ 0.95) from our molecular Bayesian analyses. We further analyzed the data using the same criteria again, but with all twelve characters ordered.

## Results

### Molecular data & phylogeny

We obtained ~3225 bp of sequences for most specimens including 36 *CTY B* (1140bp), 37 16S rRNA (974bp), 37 *BRCA1* (603bp), and 34 *APOB* (506bp) sequences. All new sequences are available in GenBank (Accession numbers: KT876727 to KT876870). No frame shift mutations or premature stop codons were observed in the coding region of any locus.

**Fig 2:**
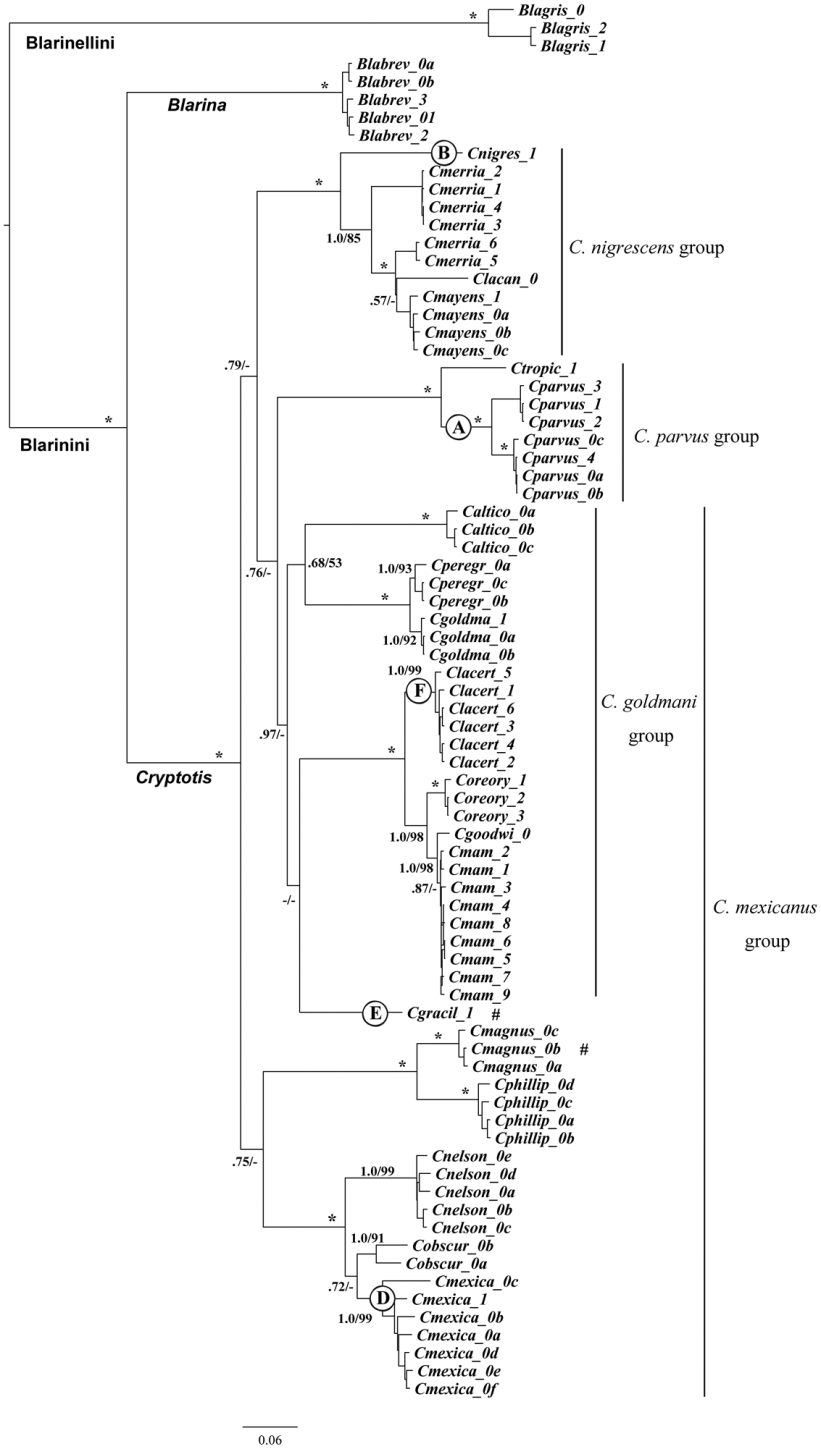
Tree resulting from Bayesian phylogenetic analyses using combined genes. Numbers at nodes indicate Bayesian posterior probabilities / ML Bootstrap values. Symbols: *, node strongly supported by both Bayesian (PP = 1.0) and ML analyses (BS = 100); –, node either not strongly supported (PP<0.5, BS <50) or not recovered. Letters on branches refer to humerus morphologies shown in Fig 1.

We used two strategies for finding the best-fitting partitioning schemes. Despite the different partitioning schemes used for BEAST (5 and 7 partitions) and ML (4 and 6 partitions), each analysis produced exactly the same tree topology. We show the Bayesian tree in Fig 2 with both ML bootstrap values and Bayesian posterior probabilities.

All individual species were supported as monophyletic with one exception: the population of *C*. *merriami* from Zacapa, Guatemala, forms a clade with *C*. *lacandonensis* and *C*. *mayensis* (PP = 1.0 and BS = 100) and is separated from the population of *C*. *merriami* in Alta Verapaz and Baja Verapaz, Guatemala, although there are no morphological differences that we could discern that distinguish the two populations.

The overall phylogenetic relationships are incompletely resolved, but seven strongly supported (PP ≥ 0.95 and BS ≥ 75) major clades were revealed. Two of the clades are congruent with species group divisions previously proposed on the basis of morphological similarity. These are the clades comprising the *C*. *parvus* group and the *C*. *nigrescens* group (Fig 2). Neither the *C*. *mexicanus* group nor the *C*. *goldmani* group is supported or strongly rejected as a clade. Seven species in the *C*. *goldmani* group (PP<0.76 and BS <50) fall into three well-supported clades, while most of the remaining species in the *C*. *mexicanus* group form two additional well-supported clades. One of these consists of the three species formerly considered subspecies of *C*. *mexicanus* [4], and the second is comprised of *C*. *phillipsii* and *C*. *magnus*. Relationships among most of the clades at higher levels are poorly resolved and characterized by relatively deep divergence and short internal branches. One exception is a potential clade that includes the *C*. *parvus* group, *C*. *gracilis*, and all of the members of the *C*. *goldmani* group. This relationship is strongly supported by the Bayesian analyses (PP = 0.97), but not by the ML analyses (BS < 50). Remarkably, this clade includes both the most ambulatory (*C*. *parvus* group) and the most fossorial (*C*. *goldmani* group) species in the genus.

### Morphological relationships

**Fig 3:**
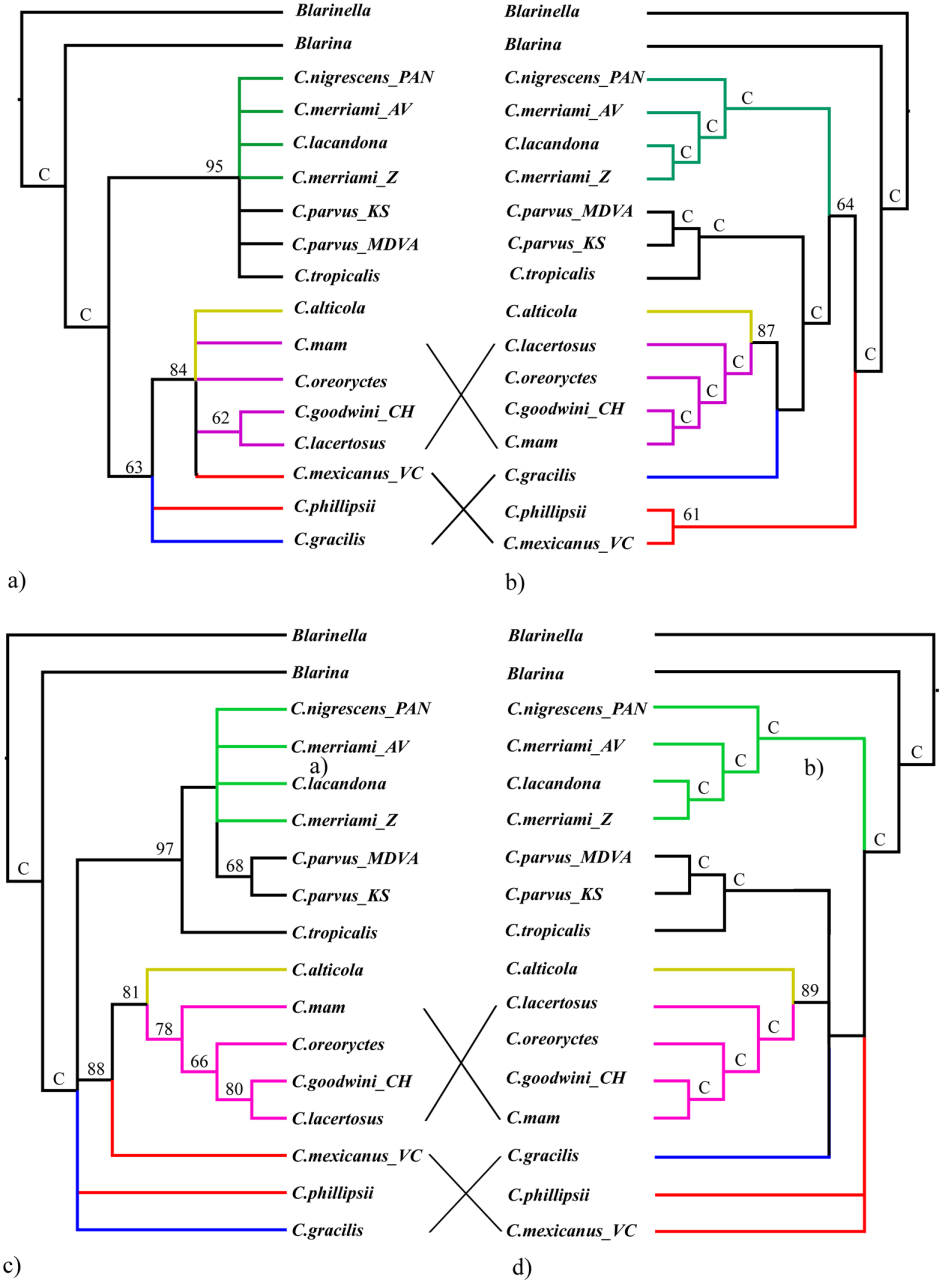
Strict consensus trees resulting from MP analyses of morphological transition series. (A) Unconstrained tree, transition series unordered. (B) Constrained tree, transition series unordered. (C) Unconstrained tree, transition series ordered. (D) Constrained tree, transition series ordered. Numbers at nodes are bootstrap values. Symbol: “C”, node constrained based on results of Bayesian analyses. Capital letters following species designations refer to geographical localities (see Table 2). Color refer to different evolutionary clades in our concatenated gene tree in Fig 1.

Analyses using unordered transition series recovered 1545 MP trees with tree lengths (TL) of 42 steps, while the analyses with ordered transition series recovered 70 trees having a TL of 50 steps (Fig 3a and 3c). When the topologies were constrained using the results of molecular phylogeny, analysis of the unordered dataset recovered a single MP tree with TL of 52, and analysis of the ordered dataset recovered 20 MP trees with TLs of 65 (Fig 3b and 3d). When only the well-supported branches are considered, the topologies of the unconstrained and constrained consensus trees were the same, regardless of whether the morphological transition series were ordered or unordered. Because of these similarities, we only discuss the trees resulting from the analyses of the ordered transition series (i.e., Fig 3c and 3d).

The unconstrained tree from the analysis of ordered transition series recovered a clade consisting of all of the species of the *C*. *nigrescens* and *C*. *parvus* groups (BC = 97). The *C*. *goldmani* species group was supported as monophyletic (BC = 78) and a sister group to *C*. *mexicanus* (BC = 81). The constrained tree, in contrast, provided a much more resolved tree, the topology of which was generally similar in structure to the molecular tree and recovered the same clades. The most striking differences between unconstrained and constrained trees were (1) the placement of *C*. *mexicanus* relative to the *C*. *goldmani* group and (2) whether the *C*. *nigrescens* group and the *C*. *parvus* group are members of the same clade.

## Discussion

### Resolution of the phylogeny

Despite the inclusion of both nuclear and mitochondrial genes in our analyses, resolution of relationships above the level of sister-species remained generally poor. Several scenarios, including rapid diversification, hybridization among lineages, and incomplete lineage sorting, could potentially be responsible for the lack of resolution [23]. Reconstruction of the mitochondrial and nuclear gene trees individually (data not shown) did not reveal any incongruences, nor did it indicate strong support for any higher relationships in the genus. These results favor a rapid diversification scenario early in the history of the genus, which is known from Late Hemphillian fossils (4.85–5.0 Mya; [24]). This scenario calls for nearly simultaneous isolation of ancestors of multiple modern clades. Given such an event, recovery of a robust and bifurcating phylogeny could be intractable, even with hundreds of additional loci [25]. This hypothesis needs to be tested using a more robust data set (e.g., ultraconserved elements [UCE] or Restriction site associated DNA [RAD]; [25]) combined with a statistic coalescent approach (e.g., Approximated Bayesian Coalescence) [26].

### Validity of species groups

Despite the unresolved higher-level relationships in our phylogenetic trees, two of the morphological species groups were strongly supported. Both the *C*. *nigrescens* and *C*. *parvus* groups are genetically cohesive associations of species, and the two groups do not appear to have a sister relationship with one another. The monophyly of the *C*. *goldmani* group is neither supported nor rejected. The topology of the molecular tree suggests that *C*. *gracilis* is embedded within this group as a sister to four species in this group (*C*. *lacertosus*, *C*. *mam*, *C*. *goodwini*, *C*. *oreoryctes*). Bayesian support for inclusion of *C*. *gracilis* within the *C*. *goldmani* group is quite high, however, neither Bayesian nor maximum likelihood analyses reject the possibility of *C*. *gracilis* being sister to the *C*. *goldmani* group.

The *C*. *goldmani* group had been considered a subgroup within the *C*. *mexicanus* group based on cranial and postcranial morphology (Table 1), but the topology of our molecular tree strongly suggests that this is not the case. The *C*. *goldmani* group appears to be sister to the *C*. *parvus* group, and these two groups together are sister to the *C*. *nigrescens* group. A well-supported clade consisting of *C*. *mexicanus*, *C*. *nelsoni*, and *C*. *obscurus* is sister to a clade consisting of *C*. *magnus* and *C*. *phillipsii*. These two clades are located on a separate branch from the *C*. *goldmani* group. Branch support values, however, are too low to be certain of these relationships, but it would take considerable shuffling to bring the *C*. *goldmani* group back into the *C*. *mexicanus* group. Thus, while the validity of the *C*. *mexicanus* group (*sensu lato*) is not rejected by the molecular phylogeny, its existence is deemed highly unlikely. The “relict” species *C*. *magnus* was not included in our morphological analyses because of a lack of readily available postcranial material, but from what we know of the morphology of its cranium and humerus [10], the sister relationship with *C*. *phillipsii* indicated by the Bayesian analyses is concordant with the morphological evidence.

Still lacking at this point is an independent assessment of the South American *C*. *thomasi* species group to determine whether it represents a monophyletic clade and how the members of this group are related to the other species groups. Inclusion of species from this group in a molecular or combined analyses may help to clarify the relationships among the other groups as well.

### Validity of species

Our analyses revealed unexpected relationships among the species included in the *C*. *nigrescens* group. Our molecular phylogenetic analyses strongly support a monophyletic clade consisting of *C*. *lacandonensis*, *C*. *mayensis*, and *C*. *merriami* from Zacapa, Guatemala, within the *C*. *nigrescens* group. Surprisingly, our results show that *C*. *merriami* from Zacapa does not form a monophyletic group with *C*. *merriami* from Alta Verapaz and Baja Verapaz, Guatemala. Despite their geographical separation, the two populations are morphologically much more similar to each other (if not identical) than to any other species, so testing of the species boundaries using comprehensive morphological and multilocus molecular data representing a wider variety of samples throughout the geographical distribution of this taxon is warranted [27]. *Cryptotis merriami* (*sensu lato*) has been recorded from 600–1650 m asl from northern highlands of Chiapas southwest to the northern Nicaragua and from isolated populations in northern Costa Rica [2, 28, 29].

### Congruence between molecular and morphological datasets

Our morphological analyses, although based on a small matrix with 12 transformation series, yielded a strong phylogenetic signal. There are strong discordances, however, between the phylogenetic trees generated with molecular vs. morphological data, particularly with the conflicting pattern of relationships of several species in the *C*. *mexicanus* group. Unconstrained morphological analyses tended to associate *C*. *mexicanus* with the *C*. *goldmani* group, yet molecular analyses showed *C*. *mexicanus* (and its close relatives) in a distinct evolutionary lineages separate from the *C*. *goldmani* group. Most of the morphological transition series we used involved the morphology of the humerus, which should be closely associated with digging ability [9]. The incongruence indicates that the morphological adaptations of the forelimbs of *C*. *mexicanus* and its close relatives (*C*. *nelsoni*, *C*. *obscurus*), which were interpreted as successful intermediate stages in the evolution of forelimb morphologies in the *C*. *goldmani* group, are more likely to be analogous convergences. Our results agree generally with the separation of the *C*. *goldmani* group from *C*. *mexicanus* and its close relatives as proposed by Guevara and Cervantes [14]. Homoplasy resulting from convergence can mislead genealogical relationships and assemble distant relatives, giving them the appearance of a clade [30]. The increase of the tree lengths in the constrained morphological analyses supports a non-parsimonious scenario for the evolution of humerus and convergent evolution toward fossorial locomotion in *Cryptotis*.

### Convergent fossorialization in Soricidae

Nevo hypothesized that the subterranean environment offered a more stable microclimate, food availability, and less predation risk as compared to open environments leading to numerous examples of convergent evolution of mammals toward fossorial locomotion [31].

Adaptive convergence towards a more fossorial existence appears to be common among shrews. Approximately 10% of living species are strongly semifossorial forms, and they are found on five continents (Africa, Asia, Europe, and North and South America) and in each of the three subfamilies of the Soricidae [32]. Comparisons of forelimb morphologies associated with fossoriality within *Cryptotis* [9] to those among shrews in the African subfamily Myosoricinae showed strongly convergent adaptions of the forelimb in the two groups [33]. In both *Cryptotis* and the Myosoricinae, much of the diversity in forelimb architecture is reflected in the morphology of the humerus. Trends include increased robustness; elongation of the humeral head; and moderate to extreme development of bony processes and other regions of muscle attachment, such as the teres tubercle and medial and lateral epicondyles (Fig 1). These findings suggest that, while there may be considerable opportunities for evolution toward a more fossorial existence within the family, the latitude for variation in forelimb morphology is strongly constrained even among subfamilies, leading to patterns of convergence that can obscure phylogeny. In the current study, we demonstrate that fossorialization can evolve repeatedly even within a single genus and at a relatively shallow time scale. It will be interesting to test whether the same constraints also exist in the Crocidurinae and in other soricine shrews, especially those from Asia (e.g., *Anourosorex*, *Blarinella*, *and Soriculus*). Fossoriality may contribute strongly to the niche diversity as well as species diversity in Soricidae, one of the largest family of mammals.

## Supporting Information

S1 Table Samples and sequences used this study. An asterisk (*) indicates the sequence was downloaded from GenBank.
(XLSX)

S2 Table Primers used for PCR and sequencing. (XLS)

S3 Table Partition schemes and evolutionary models identified using two different strategies for BEAST and RAxML analyses. In the first strategy, four genes were concatenated and defined by gene and codon positions. In the second strategy, we analyzed the partition schemes and evolutionary models for each gene separately.
(XLSX)

S1 Text Morphological Transformation Series. (DOCX)
