# Supplementary material for: Molecular Phylogeny Supports Repeated Adaptation to Burrowing within Small-Eared Shrews Genus of Cryptotis (Eulipotyphla, Soricidae)
Source: PLoS One. 2015 Oct 21;10(10):e0140280. doi: 10.1371/journal.pone.0140280 (PMC4619083; doi:10.1371/journal.pone.0140280)
Supplement: S1 Text — (DOCX) [file pone.0140280.s004.docx]

SMorphological Characters.

1. Size (condylobasal length, CBL). 0, <16.0; 1, 17-18.5; 2, 18.5-19.5; 3, 19.5-20.5; 4, 20.5-21.5; 5, 21.5-22.2; 6, ≥22.3.
2. Length of the humerus relative to the condylobasal length of the skull (hl/cbl x 100, HLR). 0, <38; 1, 38-42; 2, >42.
3. Width of the distal humerus (both epicondyles) relative to its length (hdw/hl x 100, HEB). 0, <38; 1, 39–41; 2, 41-49; 3, 50-56; 4, >56.
4. Robustness of the humerus relative to its length, indicating the ability of the humerus to resist bending and shearing stresses (hld/hl x x100, HRI). 0, <10.1; 1, 10.7-11.1; 2, 11.1–12.5; 3, >12.5.
5. Length of the teres tubercle relative to the length of the humerus along its axis of rotation (httr/har x 100, HTI). 0, <19.5; 1, 20.5-26; 2, >28.
6. Distance of teres tubercle from the proximal end of the humerus along the axis of rotation of the humerus (htt/har x 100, HTTP). 0, <38; 1, 39.5-40.5; 2, 40.6-44; 3, 44-47; 4, >47.
7. Shape of the head of the humerus in posterior aspect (SHH). 0, rounded to slightly oval, not obviously angled to slightly angled; 1, oval, long axis not parallel to axis of the shaft; 2, flattened oval, long axis not parallel to axis of the shaft; 3, elongate flattened oval, long axis not parallel to axis of the shaft.
8. Trough between head of humerus and posterior aspect of greater tuberosity. 0, shallow; 1, deep.
9. Proximally-directed process on tip of medial epicondyle. 0, absent; 1, present.
10. Distal end of pectoral ridge. 0, proximal to, or even with, middle of teres tubercle; 1, just proximal to, or even with, distal edge of teres tubercle; 2, distal to the distal edge of the teres tubercle; 3, well distal to the distal edge of the teres tubercle.
11. Protrusion of head of humerus in medial view (hh/hl x 100, HH). 0, <15; 1, 15–20; 2, >20.
12. Height of pectoral process in medial view (hpp/hl x 100, HPP). 0, <15; 1, 15–19; 2, 19–22; 3, >22.
